# Unraveling Dengue Dynamics: In-Depth Epidemiological and Entomological Analyses in Bengaluru, India

**DOI:** 10.1155/2024/7247263

**Published:** 2024-02-10

**Authors:** Vani H. C., Sujit Nath N., Jaiswal M. K., Tiwari R. P., Bharathi P., Asmita B., Shankar G., Jithen C., Pallavi V. D., Srinivas V., Ashok M., Mahamood Shariff, Renuka S., Shrinivasa B. M.

**Affiliations:** ^1^ICMR-National Institute of Malaria Research Field Unit, Bengaluru, India; ^2^Jain (Deemed-to-be-University) School of Allied Healthcare and Sciences, Bengaluru, India; ^3^JSS (Deemed-to-be-University) Academy of Higher Education and Research, Mysuru, India; ^4^Indian Air Force, Bengaluru Station, Bengaluru, India; ^5^ICMR-National Institute of Virology Bangalore Unit, Bengaluru, India; ^6^NCVBDC, Directorate of Health & FW Services, Bangalore 560023, Karnataka, India

## Abstract

In view of the increased number of detected dengue cases in Bengaluru, a request for situation analysis was received from local health authorities in the selected area. The study included epidemiological and entomological assessments to understand the same. The immature forms collected were allowed to emerge, pooled, and processed for vector incrimination. In the surveyed population (347), 20 (5.8%) reported fever cases and 12 (3.5%) were confirmed as dengue cases among the 102 families. Stegomyia indices were high. Vector incrimination studies revealed pools positive for the presence of dengue virus in flower pots, fridge trays, plastic barrels, and rubber tires habitats. This study highlights the increased risk of dengue fever incidence in communities wherepoor intra and peri-domestic sanitation practices are prevailing and recommendsregular entomological surveillance of denguevirus in its vector population..

## 1. Introduction

Dengue fever is a mosquito-borne viral infection caused by one of the four serotypes of the dengue virus (DENV) (DEN1, DEN2, DEN3, and DEN4) that are antigenically unique and closely related, which poses a growing global threat [1]. Dengue virus is majorly transmitted by two vectors, namely, *Aedes aegypti* and *Aedes albopictus* [2]. The World Health Organization (WHO) identified dengue as a significant concern, as reported cases surged from 505,430 in 2000 to 5.2 million in 2019 [3]. According to the National Center for Vector Borne Disease Control (NCVBDC), India has reported 2,33,251 dengue cases with 303 mortalities among which Karnataka accounted for 9889 cases with 9 deaths in the year 2022 [4]. Various factors including global trade, urbanization, climate change, vector suitability, and vulnerability of populations created favorable conditions for dengue transmission [5], and infections range from mild flu-like symptoms to severe complications. Diagnosis includes clinical and diagnostic RT-PCR, NS1 ELISA for early stages, and later IgM/IgG antibody tests [6]. Currently, there exists no specific vaccine or antiviral treatment, emphasizing the fact that prevention is the crucial intervention required for dengue control. The report [7] of dengue outbreaks in 12 states across the country in 2022 is an indicator of its resurgence. This spout of recently documented outbreaks saw the first case of death attributed to dengue case being recorded in the nation's capital of Delhi and a total number of 20 deaths reported in the state of Kerala (a neighboring state of Karnataka). Further dengue outbreaks have been reported in Delhi during 2023 also and certain reports [8] have correlated it to the flood-prone areas within the state. The city of Bangalore, Karnataka, has also witnessed a spike in dengue cases, with the number of confirmed cases reaching 689 in June 2023 and going up to 1589 by August 2023 [9]. In light of this increasing number of reported dengue cases in Bengaluru, a request for situation analysis was received from the local health authorities of the study site. The study included epidemiological and entomological assessments to understand the same.

## 2. Materials and Methods

### 2.1. Study Area

The selected study site called Murugeshpalya with the coordinates, 12.9581°N latitude and 77.6535°E longitude, is located in the eastern part of Bangalore city, within Karnataka state, India. It is a cosmopolitan locality, used for residential as well as commercial purposes. This area is well facilitated with schools, healthcare centers, grocery stores, restaurants, and recreational spaces. The localities surveyed within the study site (Murugeshpalya) were K R Garden, Church Street, Vinayaka Nagar, Vayu Vihar, and Ramanagari. It is a region with high population density and is made up of multicultural and multilingual populous speaking languages such as Kannada, Konkani, Tulu, Telugu, English, Kodava, Hindi, and Marwari. Situated in the heart of Bangalore, the region is at an elevation of 874 meters above sea level and is characterized by a tropical climate. The city as a whole has “rainier summers than winters and as per the Köppen–Geiger classification, the prevailing weather conditions in this region are categorized under Aw” [10]. The temperature averages around 22.9°C | 73.1°F and the average rainfall observed in a year is 960 mm | 37.8 inches. However, like other urban areas, Murugeshpalya is also facing challenges such as poor sanitary maintenance, lack of infrastructure development, high population density, and water scarcity, making the environment suitable for vector breeding and disease transmission. A combination of these factors serves as a conducive environment for vector breeding. Recently, dengue cases were reported from Vinayaka Nagar, K R Garden, Ramanagari, Vayu Vihar, and Church Street localities of Murugeshpalya, and these localities have been selected for the study based on the request received from the local healthcare authorities for an epidemio-entomological survey.

### 2.2. Study Design

A cross-sectional survey was conducted for a period of four days during the first week of August 2023. Since the list of dengue-confirmed patients was received from the healthcare authorities, epidemiological data and entomological samples were collected within 100-meter radius of each of the dengue-confirmed houses. A total of 102 households have been surveyed enumerating 347 people from the selected areas. Written informed consent was taken from the respondents. Epidemiological and sociodemographic data were collected by using a pretested, semistructured questionnaire in online Google Form. In brief, details of gender, age, travel history, and fever history from the past one month were collected from the households. Among those, households who had a fever history were further questioned for their age, symptoms, blood investigations conducted, confirmation of dengue infection, hospitalization, suspected place of mosquito bites, and current status of the disease.

### 2.3. Entomological Data Collection

**Figure 1:**
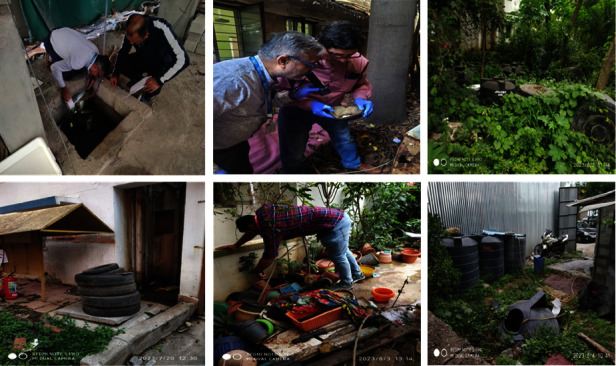
Entomological survey at intradomestic and peridomestic habitats.

Immature forms were collected from intradomestic and peridomestic habitats using standard dipping and pipetting methods [11]. All water-holding containers, including natural (tree holes, plant leaf axils, and coconut shells) and artificial (flower pots, plastic drums, tires, cement tanks, and refrigerator trays), were thoroughly inspected using flashlights when and where required (Figure 1). Where containers were found positive for immature forms, samples were collected using dippers and pipettes, and for the bigger containers, the water was sieved onto a white tray, and immature forms were subsequently collected from the sieve using pipettes. The immature forms collected were stored in a plastic container labeled indicating the date and place of collection and habitat type. All positive and negative breeding sites were recorded to calculate the larval indices. Details of houses covered, containers found, and immature breeding found in these habitats were recorded. Accordingly, the house index (percentage of houses with at least one container infested with any species of mosquito larvae and/or pupae), container index (percentage of water containers infested with at least one mosquito larva and/or pupa of any species), and Breteau index (number of containers infested with at least one mosquito larva and/or pupa of any species per 100 houses inspected) were calculated.

After collection, immature forms were brought to the insectary at ICMR-NIMR-FU, Bangalore, on the same day as the collection and were reared separately based on the habitat and study areas in controlled temperature and humidity. They were allowed to emerge into adults as per the standard protocol [12]. Adult mosquitoes were identified at the species level by using standard identification keys [13] and were pooled separately based on species, habitat, gender, and study areas. Pooled samples were stored at −80°C until further process of vector incrimination.

### 2.4. Dengue Virus Detection by Conventional RT-PCR

Vector incrimination was carried out at ICMR-NIV, Bangalore, as per the standard protocol [14]. Male and female mosquitoes were pooled separately, each pool having around 15–20 mosquitoes. Mosquitoes were homogenized using a mini hand-held homogenizer (cat#BL-MT-13K-L). Homogenized mosquito pool lysates were centrifuged at 12000*g* for 10 min to collect the supernatant for further viral RNA extraction by QiAamp viral RNA mini kit (#cat no. 52906) following the manufacturer's instructions. Extracted viral RNA was subjected to One-Step Reverse Transcription PCR to detect the dengue virus using One-Step RT-PCR Enzyme kit (#210212, QIAGEN) primers targeting 3′ UTR of DenV genome (DenV F: GGTTAGAGGAGACCCCTCCC and DenV R: GAGACAGCAGGATCTCTGGTCT). The amplified PCR products were visualized on 2% agarose gel containing GelRed Nucleic acid stain (#41003, Biotium) using the Gel documentation system (Bio-Rad).

## 3. Results

The study was conducted in different localities of Murugeshpalya, Bengaluru, in and around where dengue cases were reported. The results of sociodemographic, confirmatory dengue cases, entomological survey, and vector incrimination studies related to dengue virus transmission in the surveyed areas are discussed as follows.

**Figure 2:**
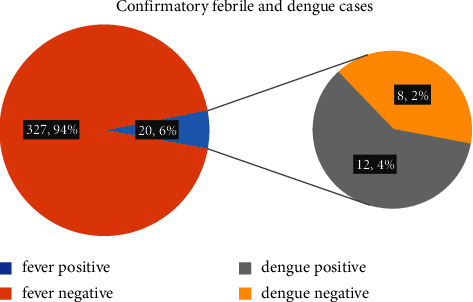
Confirmed febrile and dengue cases in the study population.

**Figure 3:**
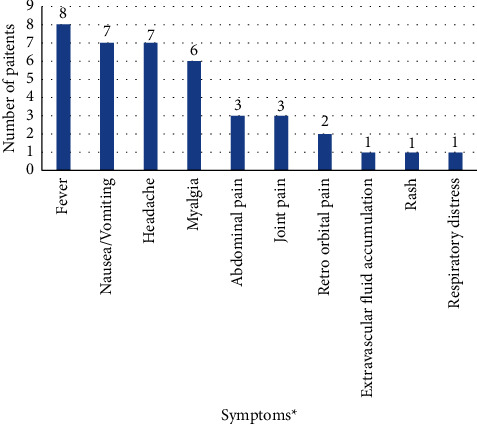
Proportion of various dengue symptoms in the study population (^*∗*^multiple responses elicited).

In the study, 102 households were covered which represent 347 population, with a male-to-female ratio of 1.18 : 1 (188 (54.2%) males and 159 (45.8%) females) and the majority of the population constitutes the age group of 18–60 (67.5%) years. Among the 102 households, 9 (8.8%) households had people with travel history in the past 30 days and 17 (16.7%) households reported the presence of fever within the past 30 days. Among them, one household with all three family members was confirmed as dengue-positive. In the surveyed population (347), 20 (5.8%) reported fever cases, and among them, 12 (3.5%) were confirmed as dengue cases (Figure 2). Among the 12 confirmed dengue cases, the median (IQR) age was 30 (9.25–33.25) years with the minimum and maximum ages being 8 and 50 years, respectively. Among them, 6 (50%) were males. Among the six females who were confirmed as dengue-positive, two were pregnant women/lactating mothers. Among the confirmed dengue cases, symptoms reported were primarily fever (12 (100%)), headache (7 (58.3%)), nausea/vomiting (7 (58.3%)), and myalgia (7 (58.3%)), and other less common symptoms included rash, joint pain, extravascular fluid accumulation, mucosal bleeding, and severe plasma leakage (Figure 3). Due to the severe form of the dengue disease, 2 (16.6%) were hospitalized. At the time of the survey, five patients (41.7%) had recovered fully, five (41.7%) were still recovering, and two (16.6%) were still experiencing symptoms. No deaths were reported. Common prevention measures taken to avoid mosquito bites were LLINs and mosquito repellents. Suspected mosquito bites as reported by the respondents were 4 (33.3%) at outdoors, 5 (41.7%) within households, and 2 (16.7%) uncertain and 1 (8.3%) at school. Among the 102 households, 27 households were found positive for pupae, and out of 174 containers examined, 52 containers were positive with the presence of immature stages of *Aedes* species. Entomological indices were calculated according to the data collected where the house index was 26.5%, the container index was 30%, and the Breteau index was 51%. The pupae-per-household index (PHI) calculated as the total number of pupae found among the total number of households inspected was 4.6 (92 pupae/collected from 20 households). The pupae-per-person index (PPI) calculated as the total number of pupae found divided by the total population of the inspected households [15] was 92/347, which is 0.27 [15].

**Figure 4:**
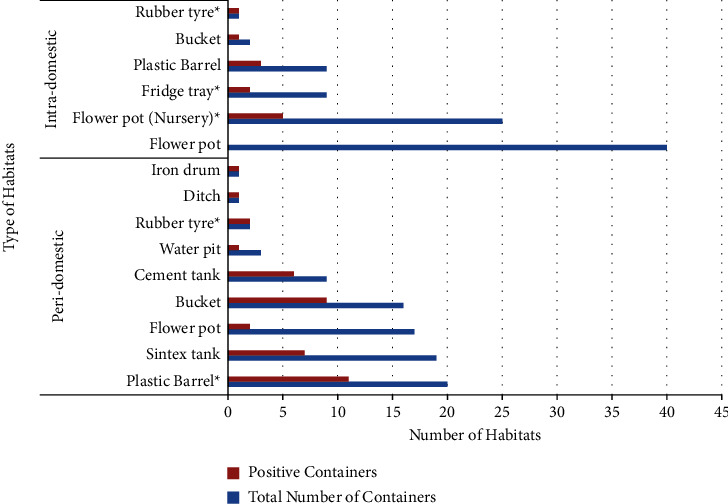
Breeding habitats of *Aedes* species examined in peridomestic and intradomestic habitats (^*∗*^positive for vector incrimination).

**Figure 5:**
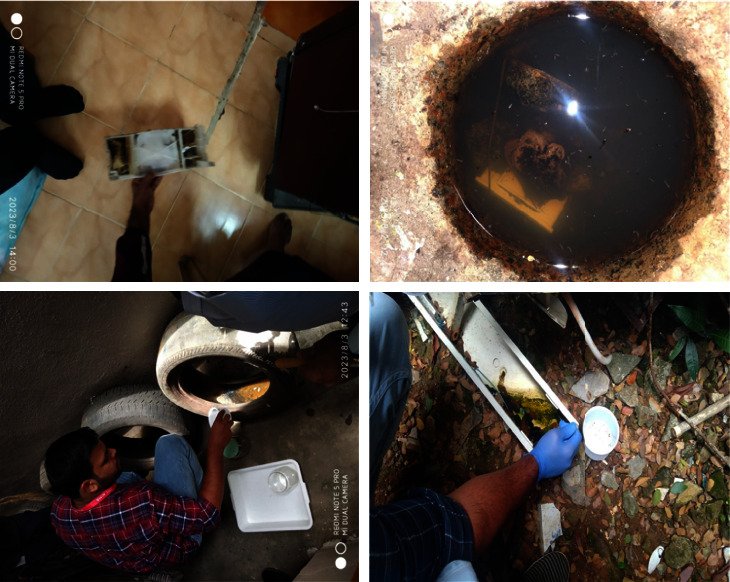
Habitat found positive for immature forms during intra- and peridomestic entomological surveys.

**Figure 6:**
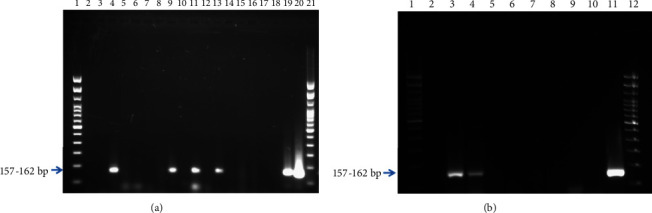
(a) Vector incrimination through PCR, amplicons at 157–162 bp. Lanes 1–21: 1 100 bp ladder, 2 fridge trays, 3 fridge trays, 4 flowerpots, 5 flowerpots, 6 flowerpots, 7 rubber tires, 8 plastic barrel, 9 fridge trays, 10 dumpyard, 11 rubber tires, 12 fridge trays, 13 plastic barrel, 14 rubber tires, 15 rubber tires, 16 flowerpots, 17 extraction negative control, 18 negative test control, 19 DENV positive control 1, 20 DENV positive control 2, and 21 100 bp ladder. (b) Lanes 1–12: 1 100 bp ladder, 2 fridge trays, 3 flowerpots, 4 rubber tires, 5 rubber tires, 6 rubber tires, 7 rubber tires, 8 rubber tires, 9 extraction negative control, 10 negative test control, 11 DENV positive control, and 12 100 bp ladder.

Based on the entomological survey conducted in the study area, 89 were peridomestic and 85 were interdomestic/intradomestic. Various *Aedes* mosquito breeding habitats were examined such as buckets, cement tanks, ditches, flower pots, iron drums, plastic barrels, rubber tires, Sintex tanks, water pits in peridomestic areas and buckets, flower pots, flower pots in nurseries, fridge trays, plastic barrel, and rubber tire in intradomestic areas. Among these breeding habitats, plastic barrels (11 (55%)) and flower pots in nurseries (5 (42%)) were found to be prominent breeding sources compared to other breeding habitats. *Aedes* mosquitoes sampled from flower pots in nurseries, fridge trays, rubber tires, and plastic barrels exhibited signs of carrying viruses (Figures 4 and 5). Cohabitation with other mosquito species of anopheles and culex immature forms was also observed in a few habitats. These immature forms collected were allowed for emergence, and among them, 307 were *Aedes aegypti* and 42 were *Aedes albopictus*. Pooled samples (22) were subjected for vector incrimination studies conducted as per the standard protocol. In the nursery out of 5 flower pot pools, 2 were tested positive similarly out of 5 fridge trays, one was tested positive, and one pool of plastic barrels and 2 pools of rubber tires were tested positive for the presence of virus (Figures 6(a) and 6(b)).

## 4. Discussion

Studies have highlighted the significance of breeding sites for *Aedes* mosquitoes as significant contributing factors for dengue virus transmission. Specific “container types” such as rubber tires and plastic barrels have been identified as high-risk breeding sites for *Aedes* mosquitoes [16]. The present study which focused on peridomestic and intradomestic areas was also able to identify flower pots (from nurseries) as a significant intradomestic breeding site. The immature forms collected from these habitats of fridge trays, flower pots, and rubber tires showed positive for vector incrimination, indicating ongoing dengue transmission in the locality. This underscores the importance of regular monitoring and cleaning of various “container types” in both (peridomestic and intradomestic) settings to mitigate the disease risk. The entomological indices such as the house index, container index, and Breteau index, recorded in the course of the study, indicate the potential for dengue virus transmission in the study site. It was also observed that the region exceeds the WHO recommended threshold for risk, thereby highlighting the need for vector control measures. These indices also throw light on the need to evaluate the intervention measures undertaken in the region. Another study conducted in Bihar, India, following the reporting of multiple dengue cases identified that urbanization, increasing construction works, conducive weather conditions, water storage practices, and migration patterns play a key role in disease spread [17]. We observed that these factors were found to be the features of the study site, which supports the cause of the dengue outbreak. site.

During the course of the survey conducted as part of the study, it was found that a large portion of subjects reporting higher frequency of mosquito bites had the presence of positive mosquito breeding sites in their intradomestic settings such as fridge trays and flower pots, in addition to breeding sites mentioned in the above studies such as rubber tires, highlighting the significance of local mosquito exposure. Together, these studies highlight the complex epidemiology of dengue fever, taking into account regional differences, societal dynamics, and clinical manifestations. As a result, they add to our understanding of the disease and can help guide targeted preventive and control strategies.

The study has identified various factors that influence vector breeding, dengue transmission, and in turn, the need for preventive measures. It is an attempt to highlight the importance of a comprehensive public health approach that encompasses repeated community health education followed by behavioral change, targeted vector control strategies, and continuous surveillance to initially reduce and then prevent dengue transmission.

The study, however, could not be conducted over an extended period owing to limitations of time set forth by the local health authorities and the need to swiftly submit a potential outbreak report to ensure timely intervention. Another challenge encountered particularly during intradomestic sampling was the difficulty in gaining access to certain residences, particularly in the more developed areas of the study site. Some of these residents cited concerns such as privacy would not allow data collection from certain households, leading to complications in the sampling process, which might have restricted the study in gaining further insights and providing a more nuanced breakup by regions within the study site.

## 5. Conclusion

The entomological survey conducted in Murugeshpalya and its surrounding areas revealed significant mosquito breeding in both peridomestic and intradomestic containers. The occurrence of confirmed dengue cases aligns with the presence of mosquitoes carrying the dengue virus. This preliminary analysis provides essential insights for targeted interventions to control the dengue outbreak in the region.

### 5.1. Recommendation

Vector incrimination studies showing denguepositivity indicated that mosquito population ofthe study site carrying dengue virus responsible for ongoing disease transmission in the community. Hence, necessary precautions need to be taken to prevent/control the outbreak. It was recommended to the concerned health authorities that the source reduction measures, peridomestic and intradomestic, be emphasized. Regular antilarval and antiadult measures are to be intensified. Health education and behavior change regarding open tanks/water storage and discarding artificial containers are to be emphasized. Containers must be properly covered up; unused containers must be discarded and weekly cleaning of containers must be performed to prevent cohabitation of species—special emphasis on fridge trays and rubber tires which were potential breeding habitats in our study.

## Data Availability

The data presented in this study are available on request from the corresponding author.
